# COVID-19 Misinformation Prophylaxis: Protocol for a Randomized Trial of a Brief Informational Intervention

**DOI:** 10.2196/24383

**Published:** 2020-12-07

**Authors:** Jon Agley, Yunyu Xiao, Esi E Thompson, Lilian Golzarri-Arroyo

**Affiliations:** 1 Prevention Insights Department of Applied Health Science, School of Public Health Bloomington Indiana University Bloomington Bloomington, IN United States; 2 Indiana University School of Social Work Indiana University Bloomington and Indiana University-Purdue University Indianapolis Bloomington/Indianapolis, IN United States; 3 Indiana University Media School Indiana University Bloomington Bloomington, IN United States; 4 Biostatistics Consulting Center School of Public Health Bloomington Indiana University Bloomington Bloomington, IN United States

**Keywords:** COVID-19, misinformation, infodemic, infodemiology, trust, trust in science, protocol, intervention, health information, prevention, behavior

## Abstract

**Background:**

As the COVID-19 pandemic continues to affect life in the United States, the important role of nonpharmaceutical preventive behaviors (such as wearing a face mask) in reducing the risk of infection has become clear. During the pandemic, researchers have observed the rapid proliferation of misinformed or inconsistent narratives about COVID-19. There is growing evidence that such misinformed narratives are associated with various forms of undesirable behavior (eg, burning down cell towers). Furthermore, individuals’ adherence to recommended COVID-19 preventive guidelines has been inconsistent, and such mandates have engendered opposition and controversy. Recent research suggests the possibility that trust in science and scientists may be an important thread to weave throughout these seemingly disparate components of the modern public health landscape. Thus, this paper describes the protocol for a randomized trial of a brief, digital intervention designed to increase trust in science.

**Objective:**

The objective of this study is to examine whether exposure to a curated infographic can increase trust in science, reduce the believability of misinformed narratives, and increase the likelihood to engage in preventive behaviors.

**Methods:**

This is a randomized, placebo-controlled, superiority trial comprising 2 parallel groups. A sample of 1000 adults aged ≥18 years who are representative of the population of the United States by gender, race and ethnicity, and age will be randomly assigned (via a 1:1 allocation) to an intervention or a placebo-control arm. The intervention will be a digital infographic with content based on principles of trust in science, developed by a health communications expert. The intervention will then be both pretested and pilot-tested to determine its viability. Study outcomes will include trust in science, a COVID-19 narrative belief latent profile membership, and the likelihood to engage in preventive behaviors, which will be controlled by 8 theoretically selected covariates.

**Results:**

This study was funded in August 2020, approved by the Indiana University Institutional Review Board on September 15, 2020, and prospectively registered with ClinicalTrials.gov.

**Conclusions:**

COVID-19 misinformation prophylaxis is crucial. This proposed experiment investigates the impact of a brief yet actionable intervention that can be easily disseminated to increase individuals’ trust in science, with the intention of affecting misinformation believability and, consequently, preventive behavioral intentions.

**Trial Registration:**

ClinicalTrials.gov NCT04557241; https://clinicaltrials.gov/ct2/show/NCT04557241

**International Registered Report Identifier (IRRID):**

PRR1-10.2196/24383

## Introduction

### COVID-19 and Misinformation

The COVID-19 pandemic has significantly affected the United States in numerous ways, both directly and indirectly. As of July 17, 2020, the number of daily new cases per 100,000 people was sharply increasing at a rate that exceeded corresponding increases in testing numbers across various states in the country [1]. The same day, the death toll due to COVID-19 in the United States was 133,600 [2], but this number did not reflect the anticipated lag time between diagnosis and fatality. In addition, there has been extensive disruption of most major societal structures, including economic and educational systems during the pandemic [3]. From July 10 to July 17, 2020, alone, the Centers for Disease Control and Prevention (CDC) reported 133 news stories about major social impact due to COVID-19 [4].

Behavioral preventive measures continue to be an effective primary public health tool for addressing challenges during the pandemic [5-7]. However, adherence to behavioral recommendations to prevent COVID-19 spread has been inconsistent, especially in the United States, with documented instances of refusal among lawmakers [8], airline passengers [9], general consumers [10], and churches [11], among others. As COVID-19 cases and related fatalities continue to rise, it is critical to identify the factors underpinning refusal to undertake basic preventive measures against disease transmission and adopt suitable measures to address them.

In addition to the spread of COVID-19, researchers have reported extensive proliferation of misinformation and conspiracies about the disease [12-14]. Intensive efforts have been made to delineate the accuracy of information about COVID-19 shared on social media (ie, by using natural language processing) [15]. However, numerous governmental and scientific organizations have simultaneously issued inconsistent and contradictory guidance about preventive measures such as face masks, further complicating the issue [16]. Although differential but reasonable interpretations of extant data and modification of recommendations in response to new data are expected of the scientific process, public correspondence, including that via official channels, has suggested otherwise. This oversaturation—with different sources of information of varying quality being constantly disseminated—adds considerably to the complexity of COVID-19 prophylaxis and may have real-world consequences. For example, one can identify specific, problematic behavioral outcomes that can be conceptually mapped to believing particular misinformed COVID-19 narratives (eg, 5G wireless, Bill Gates vaccination, and restriction of liberty) [17].

Research on misinformation and conspiracy theories, in general, has suggested that political orientation [18], religious commitment [19], and cognitive sophistication [20] are core factors associated with such beliefs. However, emerging research and commentary specific to COVID-19, including our own study, have indicated strong associations between trust in science and political and religious factors, as well as the belief in misinformed narratives and support for public health prevention efforts [17,21-24].

### COVID-19 Nonpharmaceutical Preventive Behaviors

Extant cross-sectional research investigating COVID-19 nonpharmaceutical preventive behaviors (NPBs) has frequently identified that unchangeable factors or hard-to-change factors serve as significant predictors, such as political beliefs [25,26], choice of news channel [27], age, and sex [28]. Single studies have reported that COVID-19 conspiracies may mediate between vertical individualism (believing that people are autonomous and unequal) and social distancing [29] and that beliefs about the efficacy of NPBs increase voluntary preventive compliance [30]. Other studies have also indicated that self-efficacy and perceived severity might be associative factors of NPBs [31,32].

Thus far, only 2 experimental studies on COVID-19 misinformation and NPBs have been conducted. One study focused on the likelihood of sharing false narratives on social media and found that sharing misinformation is a function of inattention, but this study did not examine trust or beliefs [33]. The other study focused on trust in science and examined political orientation as a moderating variable, using support for social distancing as the outcome, but this study did not address misinformation [23]. To our knowledge, our proposed study will be the first experimental study to examine improving trust in science as a means of reducing the likelihood of believing scientifically implausible narratives about COVID-19 and increasing intentions to engage in COVID-19 NPBs.

Specifically, we will expand on current knowledge by assessing whether exposure to a brief informational statement (in the form of an infographic) about the scientific process can increase trust in science, reduce the likelihood of believing scientifically implausible narratives about COVID-19, and increase intentions to engage in recommended COVID-19 NPBs. Given the scale and scope of the pandemic, an intervention with a small effect size, if feasible to deploy within the cost-effective US social media infrastructure, would have the potential to save lives through increased adherence to NPBs. However, it is important not to rush the deployment of brief social media campaigns without careful research and planning to verify efficacy, given that such campaigns could have iatrogenic effects even when designed by scientists and media experts [34,35]. This echoes the recent findings by Lane and Fauci [36], who remind researchers even in the context of a pandemic, it is important to generate scientifically sound evidence.

### Conceptual Framework for the Proposed Study

Our prior research on COVID-19 misinformation identified 4 different COVID-19 belief profiles [17]. The “scientific” profile was the most common (~70% of the respondents), comprising individuals who reported high believability for a statement about the zoonotic origin of COVID-19 and low believability for misinformed narratives. The other 3 “nonscientific” profiles were not named and were instead numbered alongside conceptual descriptions. Profile 2 (~8% of the respondents) comprised individuals who reported high believability for all narratives, including the zoonotic statement and misinformed statements. Profile 3 (~12% of the respondents) comprised individuals who reported moderate believability for all narratives, and the lowest believability for the zoonotic statement of any profile (although it was still reasonably high, with a mean score of 4.59 on a scale of 1-7). Finally, Profile 4 (~10% of the respondents) was similar to Profile 2, except for a comparatively lower endorsement of the narrative that 5G networks caused the spread of COVID-19. Trust in science (a scale variable, scored from 1 to 5, and computed from a 21-item questionnaire) [37] was substantively associated with different profile memberships, after controlling for political orientation, religious commitment, race and ethnicity, gender, age, and education level. Compared to the scientific profile, each 1-point decrease in trust in science was associated with 5 (for Profile 3) to 14.3 (for Profiles 2 and 4) times higher adjusted odds of belonging to nonscientific profiles [17]. Therefore, we speculate that intervening at the level of trust in science will potentially nudge some individuals into the scientific profile.

However, whether trust in science will further affect individuals’ intention to engage in preventive NPBs via mediated beliefs in misinformation remains to be clarified. To the extent that some common narratives suggest that COVID-19 does not pose a serious health threat [17], such narratives may reduce the magnitude of “perceived severity” based on the health belief model. It is also notable that cross-sectional studies have suggested an association between perceived severity, self-efficacy, and COVID-19 NPBs in Turkey and Kenya [31,32].

**Figure 1:**
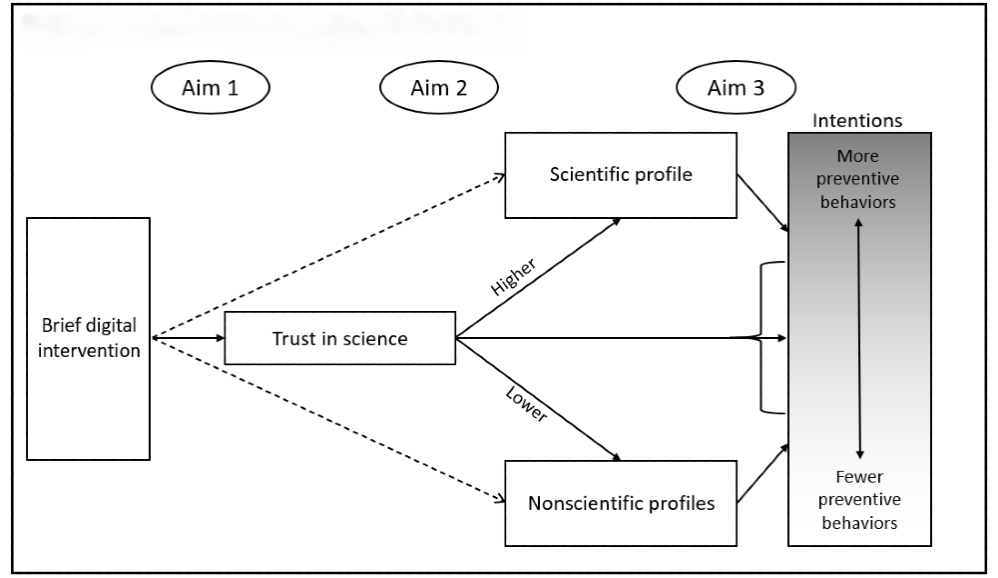
Conceptual framework of study variables.

Separately, a complex network analysis in the United Kingdom and Netherlands found that COVID-19 NPBs are most closely related to normative beliefs held by family and friends, along with the beliefs that preventive measures work [38]. Given the emerging conspiratorial narrative that face masks are an attempt to exert social control and do not actually prevent COVID-19 transmission, it is plausible that such narratives also affect the perceived efficacy of common NPBs. More direct, yet anecdotal findings in support of this mediated relationship include documented incidences of this type of misinformation being explicitly stated by individuals who publicly refuse to engage in NPBs [39-41]. Finally, in the only causal finding, Koetke et al [23] reported that trust in science exerted a direct influence on the intention to practice social distancing, but their work focused on political ideology, not misinformation, as a mediating variable. A similar associative finding by Chambon et al [38] suggested that “trust in authorities” was moderately associated with an increase in NPBs. A depiction of the overall conceptual framework of study variables is illustrated in Figure 1.

### Study Aims and Hypotheses

This study will accomplish the following 3 aims:

#### Aim 1

We aim to assess the effect of a brief informational infographic about the scientific process on trust in science. *We hypothesize that exposure to such an intervention will have a moderate, positive effect on trust in science*.

#### Aim 2

We aim to assess the effect of a brief informational infographic about the scientific process on the likelihood of believing scientifically implausible narratives about COVID-19. *We hypothesize that exposure to such an intervention will have a small, negative effect on the likelihood of believing implausible narratives, as evidenced by profile membership, and that this will be partly mediated by trust in science*.

#### Aim 3

We aim to assess the effect of a brief informational infographic about the scientific process on behavioral intentions to engage in recommended COVID-19 NPBs. *We hypothesize that exposure to such an intervention will have a small, positive effect on behavioral intentions to engage in recommended COVID-19 NPBs that will be partly mediated by misinformation profile membership*.

## Methods

### Ethics Approval and Consent to Participate

This study has been approved by the Indiana University Institutional Review Board (IRB), protocol #2008571490. Participants in both the pilot test and the main study will digitally indicate their consent to participate after reviewing a study information sheet.

### Trial Design

**Figure 2:**
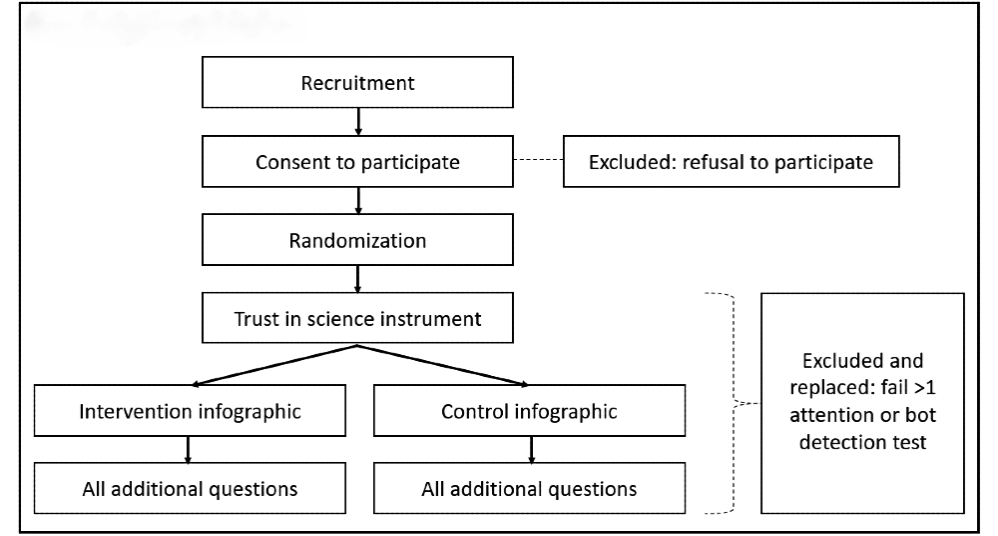
Study design and workflow.

We propose a single-stage, randomized, superiority trial comprising 2 parallel groups allocated with a 1:1 ratio. The study design and workflow involving participants is shown in Figure 2. The comparator in this study will be a control (“placebo”) infographic that is completely unrelated to science (eg, an infographic about cats) but is developed using the same communication and graphical style. The full trial, including presentation of the intervention or control condition, will be embedded within the data collection platform and will be completed in a single sitting.

### Study Setting

Participants will be recruited using the data collection platform Prolific, which is one of two primary online crowdsourced research platforms (the other is Amazon’s Mechanical Turk, or mTurk). Evidence suggests that both platforms replicate known experimental outcomes when studies are structured correctly and that the platforms compare favorably to a university-recruited subject pool [42,43]. Prolific also has the ability to collect a nationally representative sample for the United States by age, sex, and race and ethnicity, thereby improving generalizability of findings [44]. Prolific outperforms mTurk with regard to the number of accessible participants (>40,000 vs 15,000-30,000), responsiveness, diversity, and quality of participants [43,45]. Recent studies, including studies on COVID-19 perceptions, have successfully used nationally representative samples from Prolific [46].

### Eligibility Criteria

For inclusion in the study, participants must be identified by Prolific as part of a nationally representative sample. Only participants aged 18 years or older and residing in the United States will be considered. Individuals who decline to digitally sign the informed consent document will be excluded from the study and replaced by other eligible individuals. Based on best practice recommendations for crowdsourced digital research, attention checks and screens for “bots” and international users with virtual private networks to mimic US internet protocol addresses will be embedded within the instruments, and failure of more than one attention check, or any bot or location check will result in subject exclusion and replacement [47]. Attention checks will be located prior to randomization in Qualtrics, a cloud-based survey tool, so replacements will be randomized with the same allocation ratio and will be drawn in a manner that preserves the representativeness of the sample.

### Study Intervention

#### Infographic Design

The primary intervention in this study will be an infographic that is designed to build trust in the scientific process. Infographics are preferable to narratives or text because they focus on visuals as part of the storytelling process and facilitate cognitive information processing, knowledge absorption, and enhanced persuasion [48-50]. The infographic design used in this study will follow best practices in health communication. The message communicated will be simple and jargon free. The infographic will comprise visuals of individuals (scientists), charts, text, and numerical data [48]. Attention will be paid to the images used, color, frames, representation, and composition (eg, how the elements in the infographic are organized to show their relationship to each other) [49]. The design process will be completed in the following 2 stages (note that these are development stages, not trial stages).

#### Stage 1

Sample concepts and messaging will be created based on the core constructs underlying the trust in science inventory [37]. The messages will assume divergent approaches to clarify what science is, how the scientific process operates, and how science is a self-correcting process. This content may focus, for example, on how the self-correcting elements of science serve to enhance people’s quality of life (eg, addressing items 1 and 21 from the inventory [37]). In such a case, the accompanying visuals might demonstrate a flat earth progressing to a globe, and then to a picture of the earth captured from a space station. These messages will be informally discussed by the authors’ nonscientific social network in preparation for a formal pilot study.

#### Stage 2

In collaboration with professional graphic designers, the study team will design 5 infographics for the pilot testing. At present, we plan that each infographic will use an “internet comic” style of presentation that will be familiar to most participants to express core concepts underlying trust in science. The infographics will each be presented to 20 mTurk users (using the same procedures to screen for respondent quality as the overall study). Enrolled participants (N=1000) will first complete the trust in science inventory, and they will then be randomly assigned to view one of the infographics, following which they will be required to complete the inventory again. Participants will also be asked to qualitatively describe the meaning conveyed through the infographic (an open-ended question) [51] and will be asked to indicate how believable they find the infographic using a validated modification of the narrative believability scale (nbs-12) [52]. In this manner, the best-performing infographic, based on the judgment of the study team, will be used for the intervention arm of the experiment, and will be made available as a supplemental file alongside the published results. This decision will be made based on qualitative response, believability, and any observed effect on trust in science (although in the latter case, the pilot sample is not sufficiently powered to test a hypothesis, so we will consider the data broadly within the context of the other metrics).

Participants will be required to pause for at least 60 seconds while viewing the infographic (ie, the button to proceed forward will be hidden). The control infographic will be completed by the same designers but will be a placebo (ie, it will be a summary of basic information about a neutral topic, such as cats). It will mention neither science nor scientific processes.

### Primary Study Outcomes

#### Trust in Science

Participants’ “trust in science” will be measured both before and after they view the intervention or placebo using the 21-item scale developed by Nadelson et al [37]. Our previous research has found that this scale is highly reliable for diverse, online, and crowdsourced respondents [17,21].

#### Believability Profiles

Next, participants’ “believability profiles” will be computed via a latent profile analysis of the believability measures described below, which will be administered after the infographic is viewed. The measures and approach used to conceptualize believability of misinformation were developed and first used in our recent study on COVID-19 narratives [17]. As in the original study, we will not prespecify the existence of certain profiles, although we do expect to identify similar profiles in this study (potentially with differences introduced by the additional metrics described subsequently).

Response options for all believability measures will use well-established semantic differential responses for believability of different statements (eg, as used by Herzberg et al [53]), ranging from 1 (“extremely unbelievable”) to 7 (“extremely believable”). The original measures [17] assessed the believability of 4 statements related to COVID-19 that were derived from common pieces of misinformation (as of April 2020), as identified by a team at Cornell University [54]. For instance, one of the statements was, “The recent rollout of 5G cellphone networks caused the spread of COVID-19.” Another statement asked subjects about their believability of a particular statement reflecting scientific consensus (ie, the zoonotic origin of the virus). Our ability to use latent profile analysis to generate plausible and conceptually meaningful subgroups with a good model fit points toward a certain degree of validity of these questions. This is noteworthy especially given the existence of a latent profile that generally believed the scientific consensus explanation and no other narrative (which would be less likely with invalid questions). Therefore, in this study, we will assess participants’ believability of the following 6 statements, which include slightly modified versions of the 4 above-referenced statements, as well as 2 new statements based on scientific findings clarifying emergent, persistent misinformation about the use of face masks to prevent COVID-19 spread [55-57]:

- “The rollout of 5G cellphone networks caused the spread of COVID-19.”
- “SARS-Cov-2, the virus that causes COVID-19, likely originated in animals (like bats) and then spread to humans.”
- “Bill Gates caused (or helped cause) the spread of COVID-19 in order to expand his vaccination programs.”
- “COVID-19 was developed as a military weapon (by China, the United States, or some other country).”
- “The number of deaths from COVID-19 has been exaggerated as a way to restrict liberties in the United States.”
- “Wearing a face mask for COVID-19 prevention can cause oxygen deficiency or carbon dioxide intoxication.”
- “Face masks are probably not helpful in reducing COVID-19 spread in a community.”

#### Behavioral Intentions

Participants’ behavioral intentions will focus on the following specific recommendations current proposed by the CDC [58]:

- Wash your hands often (or use a hand sanitizer that contains at least 60% alcohol).
- Avoid close contact (stay at least 6 feet from other people).
- Cover your mouth and nose with a mask when around others.
- Cover coughs and sneezes.
- Clean and disinfect frequently touched surfaces daily.
- Monitor your health daily.

Each recommendation will be placed into a behavioral intention questionnaire format to assess self-reported likelihood of behaviors using a guide published by Azjen who proposed the Theory of Planned Behavior [59]. For example, “I intend to clean and disinfect frequently touched surfaces daily for the next month,” with response options ranging from 1 (“likely”) to 7 (“unlikely”). These questions will be administered after the infographic is viewed.

### Covariates

We will also collect the following additional variables to serve as covariates in the models based on other factors suspected to influence trust in science, believability of misinformation about COVID-19, and/or behavioral intentions regarding NPBs.

- Political orientation and religious commitment, using the scales from our previous works [17,21]
- Race and ethnicity, gender, age, and education level
- Whether the respondent has been diagnosed with, or believes they have had, COVID-19 [60]
- Perceived severity and self-efficacy regarding COVID-19, based on the health belief model and that used by Yıldırıma & Gülerc [31] (which was derived from similar work related to SARS)
- Normative beliefs about friends’ and family’s COVID-19 behaviors (single item from Chambon et al [38])

### Sample Size

We will recruit 1,000 individuals using Prolific, which is the maximum sample size permitted for a nationally representative sample via this platform. This sample size will give allow us to detect small (Cohen’s d=0.18) differences between both groups with 80% power. This effect size would be more than sufficient for both analysis types, that is, LMM (linear mixed models) and path analyses, thus accounting for power and other sample size considerations common to path analysis (eg, overspecification) [61].

### Recruitment

Participants will be recruited via the Prolific platform to ensure a nationally representative sample of the US is composed with regard to age, sex, and race and ethnicity. To ensure compliance with institutional review board requirements, the questionnaires and procedures (including the study information sheet) will be hosted securely within Indiana University’s Qualtrics platform. Prolific will refer the sampled individuals to the Qualtrics system. Thereafter, we will randomly generate a unique identification for each Qualtrics user and ask the participants to enter it into Prolific to verify that they have completed the survey. We anticipate the survey will take no more than 15 minutes to complete; compensation will be set by Prolific to be equitable but noncoercive for research. The proposed methodology mirrors the protocol we successfully used with mTurk in our previous works [17,21].

### Allocation of Interventions

The allocation sequence will be managed using the Randomizer tool in the Qualtrics platform [62] to ensure a 1:1 allocation of participants to intervention (1) and control (0) arms, each comprising 500 participants. Allocation will occur after consent has been processed. Furthermore, the procedure is automated, thereby ensuring allocation concealment.

Although this is, in practice, a double-blind study since participants will be unaware that they are randomized and all study mechanics will be processed using a computer, analysts will not be blinded to the meaning of the assignment variable. However, 2 independent consultant analysts have been retained to verify all results and subsequent interpretation.

### Data Collection and Management

All data will be collected and stored using the QualtricsXM (Qualtrics) digital platform. This platform enables direct exporting of data to a variety of formats (eg, Excel and SPSS). To promote data quality, respondents will be required to respond to all items on each page of the survey before proceeding to the next part of the study, which would ensure there are no missing data from completed cases. While excellent participant retention is expected since the study (including intervention) is brief and occurs contemporaneously, some participants may decide to quit prior to completion. If a participant has provided any data beyond the study information sheet, the case will be analyzed according to the study arm to which it was assigned, with missing data managed as described in Statistical Analysis (eg, intention-to-treat). However, this will not apply to participants who are excluded due to failed quality checks (see Eligibility Criteria) because these are designed to filter for cases that were not eligible but were enrolled in the study inappropriately (eg, “bots” or individuals who mask their global location with a virtual private network).

### Statistical Analysis

#### Missing Data and Data Quality

Missing data will be addressed using either full information maximum likelihood or Markov Chain Monte Carlo multiple imputation strategies [63]. In case there is a violation of missing at random in preliminary analyses (which is unlikely), we will incorporate strategies representing the missingness. We will further explore data quality in terms of outliers, measurement error, non-normality, and variance heterogeneity. Robust methods of analysis (eg, Huber-White robust standard errors) will be used, as appropriate [64]. For all multi-item measures, we will evaluate reliability prior to computation of the variable.

#### Analyses

The following analyses will be performed for the 3 aim statements:

**Aim 1**: We will use an LMM to examine the effect of the intervention on trust in science, controlling for specified covariates (see Covariates).

**Aim 2**: We will first examine the profiles of believability in our control and intervention group using latent profile analyses [65,66]. After determining the number of classes and identifying scientific versus nonscientific profiles, we will conduct path analyses linking the brief digital intervention to believability profiles and the mediator (trust in science).

**Aim 3**: We will first examine the outcome variables for Aim 3—intention to engage in NPBs. Although we expect these intention-based items to function as a monotonic scale that can be collapsed into a single variable, we will need to conduct exploratory factor analysis to determine whether this is the case. The number of factors identified will affect how this outcome is treated in the subsequent part of Aim 3.

We will also examine the mediation effect of believability profiles on the association between behavioral intentions, trust in science, and the primary outcome for Aim 3 (ie, intention to engage in NPBs) in the post-test. Next, we will conduct path analyses linking the brief digital intervention to believability profiles and the mediators (trust in science and believability profiles), and behavioral intentions in the post-test.

For both Aims 2 and 3, we will use model fit statistics (eg, root mean square error of approximation, comparative fit index, and Tucker-Lewis Index) and examine specific indices of ill fit (eg, modification indices). To increase power and maintain Type I error, we will adapt the posterior probability method (eg, partial *P* value) for formal mediation analyses with intervening variables [67]. We aim to elucidate key mediational chains between our mediators (ie, trust in science and believability classes), predictors (ie, behavioral intervention statement), and outcome (ie, intention to engage in NPBs) through this design, instead of simply looking at the link between the intervention and intention to engage in NPBs.

## Results

This study was funded in August 2020, approved by the Indiana University Institutional Review Board on September 15, 2020, and prospectively registered with ClinicalTrials.gov. We expect the infographics to be finalized in early December 2020 and plan to run the pilot test in the same month. The entire experiment should be completed, and the results published, in 2021. This protocol was submitted as prepared for review in a grant format to the Indiana Clinical and Translational Sciences Institute in June 2020. Aside from stylistic modifications made in transforming the grant into a paper, and in addressing grant reviewers’ and manuscript reviewers’ comments, the protocol reflects the originally proposed work.

## Discussion

### Next Steps

The primary purpose of this study is to test an actionable, brief digital intervention that can modify trust in science and thereby mitigate belief in misinformation and increase behavioral intentions to engage in NPBs. Thus, if any hypotheses are validated, the primary next step will be to disseminate this information and coordinate use of the infographic. Two articles published in *Nature* [68,69], (including one by that journal’s editorial board), acknowledged the need for researchers to honestly and transparently address COVID-19 misinformation, and to specifically look into trust, but the cited research primarily examined networking and spread dynamics, not prevention.

### Data Monitoring, Interim Analyses, and Auditing

This study will not have a data monitoring committee because data collection will be automated, stored, and archived. Moreover, no harms are anticipated to occur as a result of the experiment. No interim analyses are planned. Data and analyses will be reviewed by all members of the study team as well as by independent statistical consultants.

### Study Limitations

This study has several limitations that might affect its conclusions. First, the data are based on self-reporting. For believability and trust in science, this is a less substantive issue; however, it is potentially subject to social desirability bias, which we will attempt to minimize. Although extant behavior models like the Theory of Planned Behavior [59] indicate that intentions are direct antecedents to behavior, we cannot be certain that intentions will result in actual behavior. Nevertheless, measuring this would be beyond the scope of this protocol. Second, we are unable to control the conditions in which people will participate, but we will incorporate attention checks throughout the process. Furthermore, prior studies have been able to replicate known experimental findings using crowdsourced digital participants, suggesting that the undue influence of inattention can be minimized [42,43]. Third, this experimental approach is likely subject to a Type II error because a real-life application of this process would entail repeated exposures over time, whereas this approach tests the effect of a single exposure. Fourth, other variables that might influence behavioral intentions also likely exist, but by recruiting a large sample and randomizing participants, we will be able to more cleanly isolate the differences attributable to the intervention.

Finally, generalizability to individuals who do not have internet access or who are unable to participate in online surveys might be limited. However, regarding the concern about access, it is noteworthy that approximately 90% of US adults used the internet in 2019, with higher percentages reported for younger adults and lower percentages, for older adults. Of note, 73% of individuals aged >65 years reported they had internet access in 2019 (although it began trending sharply upward in 2018) [70]. Furthermore, as noted throughout our proposal (and in academic papers published since submission [71]), social media remains a primary source of misinformation about COVID-19. Considering that the online data collection platform introduces bias as a result of internet access, we speculate that controlling for age (which we already do) may attenuate this concern to an extent, in addition to the fact that the intervention itself is designed for distribution on social media.

Regarding the latter point (opt-in participation), we acknowledge that individuals who opt into survey completion may differ systematically in one or more ways from those who do not. However, the data collected through platforms like Prolific are of good quality and have replicated results of multiple established experiments, thereby reducing concerns about the influence of opt-in participation on the results. Further, studies on similar crowdsourced survey platforms such as mTurk have found that the study participants are similar to the overall US population with regard to a number of different characteristics. However, they also found that survey takers are younger and are more educated, on average, than the US population as a whole [72-74]. Both variables (ie, age and education level) will be controlled in our study’s analyses. This further mitigates concerns that the participants recruited by Prolific are systematically different from those that might be enrolled in a similar experiment in other circumstances.

## Appendix

Multimedia Appendix 1 Review of original grant submission (Reviewer 1, scores redacted by funder).

Multimedia Appendix 2 Review of original grant submission (Reviewer 2, scores redacted by funder).

## Abbreviations

CDC: Centers for Disease Control and Prevention
LMM: linear mixed model
mTurk: Amazon Mechanical Turk
NPB: nonpharmaceutical preventive behavior
