# Supplementary material for: COVID-19 Misinformation Prophylaxis: Protocol for a Randomized Trial of a Brief Informational Intervention
Source: JMIR Res Protoc. 2020 Dec 7;9(12):e24383. doi: 10.2196/24383 (PMC7722482; doi:10.2196/24383)
Supplement: Multimedia Appendix 1 [file researchprotocols_v9i12e24383_fig.pdf]

COVID-19 Pilot Research Application
Review Template

Principal Investigator(s): Jon Agley

Title of Application: Pilot Study of a Brief Informational Intervention for COVID-19 Misinformation Prophylaxis

The NIH scoring system defined below should be used for the scored criteria and the overall impact score (use only integer scores, no decimals).

Impact	Score	Descriptor	Additional Guidance on Strengths/Weaknesses
High	1	Exceptional	Exceptionally strong with essentially no weaknesses
	2	Outstanding	Extremely strong with negligible weaknesses
	3	Excellent	Very strong with only some minor weaknesses
Medium	4	Very Good	Strong but with numerous minor weaknesses
	5	Good	Strong but with at least one moderate weakness
	6	Satisfactory	Some strengths but also some moderate weaknesses
Low	7	Fair	Some strengths but with at least one major weakness
	8	Marginal	A few strengths and a few major weaknesses
	9	Poor	Very few strengths and numerous major weaknesses
**Minor Weakness:** An easily addressable weakness that does not substantially lessen impact<br>**Moderate Weakness:** A weakness that lessens impact<br>**Major Weakness:** A weakness that severely limits impact			

OVERALL IMPACT

Reviewers will provide an overall impact score to reflect their assessment of the likelihood for the project to establish a sustained research program or significant new IP, in consideration of the following five scored review criteria, and the additional unscored review criteria. An application does not need to be strong in all categories to be judged likely to have major scientific impact.

Overall Impact	Score (1-9):

Strengths

- Timely cost-effective project
- Addressing a growing problem that, if demonstrated to be effective, could affect not only actions related to COVID-19 but other science-based issues as well

Weaknesses (Please categorize Major vs. Minor)

- Need clearer explanation of how the infographic will be designed and piloted before studying

- Need clearer explanation of control and intervention messages to assess potential effect on knowledge checks

SCORED REVIEW CRITERIA

Reviewers will consider each of the five review criteria below in the determination of scientific and technical merit, and give a separate score for each.

1. Significance	Score (1-9):

Strengths

- Timely topic that could demonstrate cost effective outcomes
- Creating evidence-based communication tools in response to calls from academic and public health professionals to avoid perpetuating existing information problems

Weaknesses (Please categorize Major vs. Minor)

-

2. Investigator(s)	Score (1-9):

Strengths

- Previous studies in this area
- Worked together on previous studies

Weaknesses (Please categorize Major vs. Minor)

-

3. Innovation	Score (1-9):

Strengths

- Novel area of exploration within the topic of COVID-19
- Ultimate output will be disseminated via social media platforms

Weaknesses (Please categorize Major vs. Minor)

- Does not address already recognized influencing factors (need to address how those other factors noted in the proposal will be assessed, possibly controlled for, etc.)

4. Approach	Score (1-9):

Strengths

- Nationally representative survey using validated recruitment strategies
- Theoretically grounded survey constructs (HBM, TPB)

Weaknesses (Please categorize Major vs. Minor)

- No detailed discussion of how the infographic will be developed and tested before use (simply states 5 experts will give advice on wording and appearance)
- Unclear what the control and intervention communication tools will communicate and how those might influence responses

5. Environment	Score (1-9):
**Strengths**<br>• Previous studies support the use of Prolific as a nationally representative recruitment tool<br>**Weaknesses (Please categorize Major vs. Minor)**<br>• Online data collection will not represent individuals who do not have Internet access or participate in online surveys	

OTHER CRITERIA (NOT SCORED)

As applicable for the project proposed, reviewers will consider the following additional items in the determination of scientific and technical merit, but will not give separate scores for these items**.** Although these criteria will not be evaluated with a specific score, the overall evaluation of the application should take these factors into account.

Guidelines
Comments (Required Unless Not Applicable):<br>•

Budget and Period of Support
Recommended budget modifications or possible overlap identified:<br>• More details need to be given about what the expert panel members will actually do when assisting in the development of the communication tool (and how that equates to $250)

Protections for Human Subjects
Comments (Required Unless Not Applicable):<br>• Mentioned following IRB guidelines

Vertebrate Animals
Comments (Required Unless Not Applicable):<br>• n/a

Biohazards
Comments (Required Unless Not Applicable):<br>• n/a

Resubmission
Comments (if applicable):

• n/a

ADDITIONAL COMMENTS TO APPLICANT

Reviewers may provide guidance to the applicant or recommend against resubmission without fundamental revision.

Additional Comments to Applicant (Optional)
•
